# Home Health and Community Care Workers’ Occupational Exposure to Secondhand Smoke: A Rapid Literature Review

**DOI:** 10.1093/ntr/nty226

**Published:** 2018-10-26

**Authors:** Kathryn Angus, Sean Semple

**Affiliations:** Institute for Social Marketing, Faculty of Health Sciences and Sport, University of Stirling, Stirling, Scotland

## Abstract

**Introduction:**

Although many workers are protected from exposure to secondhand tobacco smoke (SHS), home health and community care workers enter domestic settings where SHS is commonly present. Little is known about the extent of SHS exposure among this occupational group.

**Methods:**

A rapid review to examine the literature on home health and community care workers’ exposure to SHS at work and identify research gaps. Systematic searches combining terms for SHS exposure (eg, “tobacco smoke pollution”) with terms for home health and care workers, patients and settings (eg, “home health nursing”) were run in CINAHL and Medline (with no date or language limitations). Web site and backward-forward citation searches identified further papers for narrative review.

**Results:**

Twenty relevant publications covering seventeen studies considered home health or community care workers’ exposure to SHS either solely or as part of an assessment of other workplace hazards. Eight studies provided data on either the proportion of home care workers exposed to SHS or the frequency of exposure to SHS. No studies provided quantification of SHS concentrations experienced by this group of workers.

**Conclusions:**

Exposure to SHS is likely to be common for workers who enter private homes to provide care. There is a need for research to understand the number of workers exposed to SHS, and the frequency, duration, and intensity of the exposure. Guidance should be developed to balance the rights and responsibilities of those requiring care alongside the need to prevent the harmful effects of SHS to workers providing care in domestic settings.

**Implications:**

Very little is known about home health and community care workers’ exposure to SHS. There is a need for research to quantify how many workers are exposed, how often and for how long exposure occurs, and the concentrations of SHS experienced. In many countries, home health care workers may be one of the largest working groups that experience exposure to SHS as part of their employment. The public health community needs to engage in a debate about how home health care workers can be best protected from SHS.

## Introduction

The introduction in many countries of smoke-free legislation in enclosed public spaces has helped protect workers from exposure to secondhand tobacco smoke (SHS).^1^ Where smoke-free laws have been implemented there tend to be some exemptions and, as a result, some occupational groups continue to be exposed to SHS as part of their daily activity. Recent data from the United States have indicated that workers in service and “blue collar” industries are considerably more likely to be exposed to SHS than those in “white collar” occupations.^2^

Home health and care workers (including Home Health Care Professionals or Community Staff) are likely to be one of the largest remaining occupational groups that continue to experience frequent and high concentrations of SHS as part of their work activity. There are over 600 000 workers employed in this sector in the United Kingdom,^3^ over 2 million in the United States^4^ and growing numbers globally carrying out nursing and care tasks that involve entering private residences where smoking is unrestricted by law. The workforce is predominantly female and estimates suggest the number of workers employed in this sector are likely to increase as life expectancy rises and elderly people are cared for within their own home.^5^

Current guidance and policy measures used by those providing health and social care to assess and manage the risks to the health of home health care workers from SHS are fragmented, and often poorly understood and managed. Guidance prepared by the UK Royal College of Nursing (RCN) in 2006 on protecting community staff from exposure to SHS provides recommendations for best practice for staff and managers – although this is now listed by the RCN as “use with caution” due to the fact that it has not been reviewed for over 10 years and may no longer be fully applicable.^6^ That guidance suggests educating patients about the need to provide community staff with a smoke-free space and for the patient not to smoke during a home visit. The guidance also advises that patients should ensure the area of the visit has been smoke-free for 1 hour before the visit. This now conflicts, to some extent, with more recent public health messages such as the “Right Outside” campaign in Scotland that have used the message that SHS remains in air for up to 5 hours after a cigarette is extinguished.^7^

Anecdotal evidence suggests that exposure to SHS is a real concern for workers in the home health and community care sector and, with recent and planned measures to protect prison staff from exposure to SHS,^8^ those involved in home visits feel left behind in terms of health protection to a known carcinogen.

Given that Article 8 of the Framework Convention on Tobacco Control calls for protection of all “citizens from exposure to tobacco smoke in workplaces” and the ongoing development of smoke-free regulations globally, we conducted a rapid review of the literature to identify studies that have considered home care workers’ exposure to SHS. We also aimed to identify research gaps to stimulate further discussion.

## Methods

A rapid literature review was carried out to identify publications that have considered home health care workers’ exposure to SHS. Rapid reviews are an increasingly used approach to scope the existing research by streamlining the systematic review process and providing meaningful data to inform policy and practice decisions.^9,10^ Employing this methodology, two academic databases were searched (CINAHL and Medline with no date or language restrictions) on February 12, 2018 using a strategy that combined terms for SHS exposure (eg, “tobacco smoke pollution” OR “air pollution, indoor” OR secondhand smoke OR passive smoking) with terms for home health and care workers, patients, and settings (eg, “home health nursing” OR “house calls” OR “nurses, community health” OR housebound OR private dwelling; see Supplement for the full Medline strategy). Additional internet searches using key search terms from the search strategy were made of the Bielefeld Academic Search Engine (BASE), Google.co.uk, and the British Library’s Electronic Table of Contents (ZETOC) to identify additional relevant academic literature. Searches for the included publications’ citing articles (via Web of Science) and cited articles were also carried out.

The review excluded dissertations and studies of workers who are solely based in residential care homes where smoking is generally controlled by legislation or owner rules, and instead focused on workers who are required to enter private domestic settings as part of their role. This included home health care professionals, home care nurses and aides, domiciliary care workers, community nurses, midwives, and early intervention workers. Although acknowledging that some health care facilities still permit smoking or have poor enforcement of smoking restrictions, and home health and community care workers operating from these buildings may experience nontrivial exposure to SHS in these settings, the review was restricted to considering exposure to SHS that occurred in private homes. The review was also restricted to those providing health care or assistance and excluded worker groups who visit homes to carry out maintenance, inspections, or delivery. Search results were screened for relevance by one author initially (KA), with both authors reviewing the final results for inclusion. Screening involved removing those papers where there was no consideration made of home care and/or community care workers’ exposure to SHS. This was primarily carried out at the level of title and abstract but where these were ambiguous screening occurred at full text level. For studies identified as containing relevant information, data were extracted by one author (SS) and checked for accuracy by the second (KA). Where disagreements were identified, these were resolved by rechecking the paper and discussing the relevant text.

The review considered the fidelity of the methods used to assess workers’ exposure to SHS. This was achieved by listing the method used and using three questions from the US National Institute for Health Quality Assessment Tool for Observational Cohort and Cross-Sectional Studies relevant to exposure assessment methods.^11^ These were:

1. For exposures that can vary in amount or level, did the study examine different levels of the exposure as related to the outcome (eg, categories of exposure, or exposure measured as continuous variable)?
2. Were the exposure measures (independent variables) clearly defined, valid, reliable, and implemented consistently across all study participants?
3. Was the exposure(s) assessed more than once over time?

## Results

**Figure 1.**
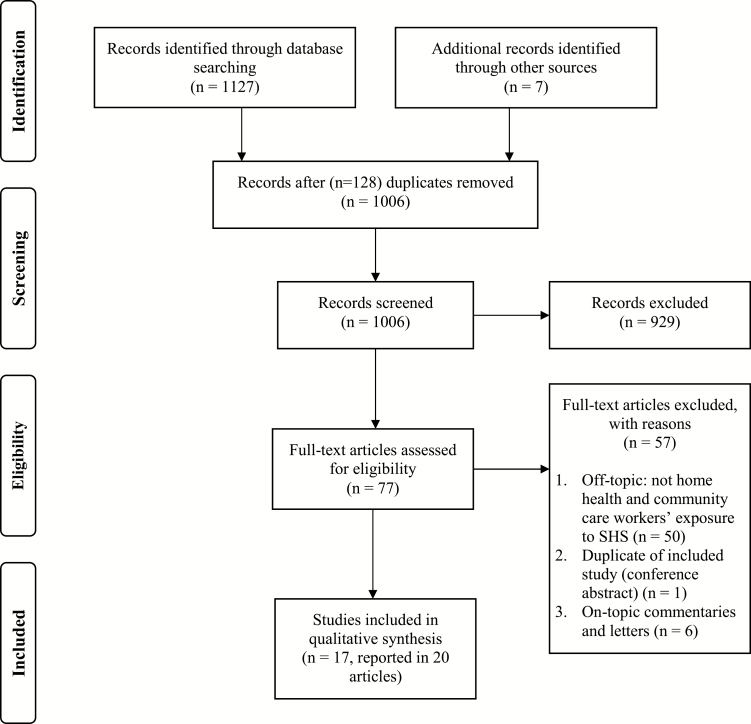
PRISMA flow diagram of identification and selection of studies

From 1134 publications identified in the search, 20 publications in academic and peer-reviewed practitioner journals were deemed eligible for inclusion in the review. Figure 1 provides a PRISMA flow diagram of identification and selection of studies. The 20 papers^12–31^ described 17 studies; one study was described across three papers^25–27^ and a further study was described in two publications.^28,29^ Studies are listed in Table 1 with brief details of the country, size (the percentage of female workers), and evidence of SHS exposure as reported within the study. Of these seventeen studies, six involved qualitative research including semi-structured one-to-one interviews and/or focus groups with home health or community care workers^13,17,19,21,22,30^; seven were cross-sectional surveys^12,15,16,18,20,25,31^; two were mixed method studies involving a survey and additional semi-structured interviews with a sample of respondents^22,28^; one was a case report^14^; and one was development of a training simulation for workers.^24^ Twelve of the studies were from the United States, two from Canada, and one each from Australia, Denmark, and Sweden.

**Table 1.** Studies Identified in the Review

Study	Location	Study type	Size (% female)	Evidence of SHS exposure
Stephany, 1993^12^	USA	Cross-sectional questionnaire of home care nurses.	*N* = 20 (NR)	Potential for personal harm from environmental agents such as SHS was a concern.
Markkanen et al., 2007^13^	USA	Qualitative study of focus groups with home health care nurses and aides.	*N* = 24 (NR)	SHS was noted under “hygiene issues” in terms of general work hazards described in the focus groups.
Gehrs et al., 2008^14^	Canada	Case study: Ethical and clinical deliberations on protecting workers from SHS.	*N* >1^a^ (NR)	This case study is of a real-life scenario of a community mental health team’s home visits to a smoking client and considers the client and staff rights and responsibilities.
Gershon et al., 2008^15^	USA	Cross-sectional questionnaire of home health care registered nurses.	*N* = 738 (95%)	72% reported experiencing exposure to SHS as part of their job.
Sherman et al., 2008^16^	USA	Cross-sectional questionnaire of home health care aides.	*N* = 823 *(93%*)	31% reported experiencing exposure to SHS as part of their job.
L’Heureux, 2009^17^	USA	Qualitative study of interviews with home care nurses (*n* = NR) and a focus group with nurses who work in a therapeutic group home (*n* = 4).	*N* = 4 (NR)	Nurses were highly concerned about their exposure to SHS in home care and group home settings and were reluctant to take action to minimize their exposure due to their role as advocates for their patients’ and clients’ rights.
Nabe-Nielsen et al., 2009^18^	Denmark	Cross-sectional questionnaire of female health care workers in the elder-care sector.	*N* = 4590 (100%)	Exposure to SHS reported as 24% in night workers, 28% in evening workers and 31% in day workers.
Berg et al., 2012^19^	Sweden	Qualitative study of focus groups with female nurses involved in home visits.	*N* = 15 (100%)	Interviews identified four key themes: nurses placing the smoking care-recipient first; putting their own health and wellbeing at risk; feeling abandoned by their employer; and finding solutions in everyday home nursing care.
Keske et al., 2013^20^	USA	Cross-sectional questionnaire of home-visitation workers attending a conference.	*N* = 316 (NR)	Three survey domains: SHS exposure; use of SHS mitigation strategies; and knowledge of agency rules. 83% reported exposure to SHS; median exposure 3.5 h per month.
Markkanen et al., 2014^21^	USA	Qualitative study of interviews and focus groups with home health care nurses and aides.	*N* = 99 (93%)	Indoor air quality issues, including smoking of clients had a high citation frequency both in focus groups and individual interviews.
Polivka et al., 2015^22^	USA	Mixed-methods study (questionnaire, focus groups, and individual interviews) of hazards encountered by home health care providers in client homes.	*N* = 68 (95%)	Poor air quality reported as a hazard by 87% of workers. SHS and inadequate ventilation were noted as the major causes of poor air quality.
Terry et al., 2015^23^	Australia	Qualitative study of interviews with community nurses.	*N* = 15 (87%)	Smoking was raised as a significant concern. Participants agreed that SHS is dangerous for their health and is an issue that remains difficult to overcome.
Darragh et al., 2016^24^	USA	Development of a gaming simulation as health and safety training for home health care workers.	NA	Simulations include exposure to SHS within home settings.
Hittle et al., 2016^25–27^	USA	Cross-sectional study involving one-to- one interviews with home health care nurses and aides^25,27^ and also home health care physical and occupational therapists.^26^	*N* = 58 (89%,^25^ 90%,^26^ 86%^27^)	Participants asked about exposure to SHS; average exposure was 95–284 h per annum; also converted to a daily figure later in the paper (15–46 min per day).^25^ Exposure to SHS average of 236 times per year.^26,27^
Quinn et al., 2016^28,29^	USA	Cross-sectional questionnaire about home care aides’ occupational safety and health experiences,^28^ qualitative feedback on findings of^28,29^.	*N* = 1249 (87%)^28^; *n* = 84 (NR)^29^	Home care aides reported that 9.9% of visits involved a client who smoked indoors.^28^ SHS was rated as an important health and safety concern.^29^
Wills et al., 2016^30^	USA	Qualitative study of semi-structured interviews and focus group discussions with home health care personnel.	*N* = 68 (95%)	Identified hazards of exposure to SHS.
Wong et al., 2017^31^	Canada	Cross-sectional survey of home care nurses.	*N* = 823 (95%)	Self-reported (“regularly” or “always”) exposure to SHS: 44.8%.

SHS = secondhand smoke; NR = not reported; h = hours; NA = not applicable; min = minutes.

^a^Paper refers only to the “Assertive Community Treatment (ACT) Team.”

Eight studies provided limited data on either the proportion of home health or community care workers exposed to SHS or quantification of the level or frequency of exposure to SHS experienced by this group.^15,16,18,20,22,25,28,31^ The largest reported work in this area was a questionnaire study of 4590 female health care workers in the eldercare sector in Denmark.^18^ This work showed approximately 24%–31% of workers reported exposure to SHS during their work shift. A similar proportion (31%) of home health care aides in New York reported exposure to SHS as part of their job.^16^ A study from Canada indicated that 45% of 823 home care nurses reported being exposed to SHS either “regularly” or “always,”^31^ whereas a cross-sectional study of home visitation, early intervention workers from the United States described that over 80% experienced exposure to SHS during their work in homes.^20^

There was very little data available on either frequency or duration of exposure. A qualitative study of 54 home health care nurses and home health care aides in the United States found that average duration of exposure was between 95 and 284 hours per year.^25^ A large study (*n* = 1249), set in the United States, found that almost 10% of home visits carried out by home health care aides involved a client who smoked indoors.^28^

One study highlighted key themes: care workers placing the smoking care recipient first and thus putting their own health and well-being at risk, feeling their employer did not take the issue of SHS exposure seriously, and finding practical solutions in everyday home nursing care.^19^ This was also reflected in another study which showed that only 14% of home visitation workers in the United States reported knowledge of rules at their organization to request that clients do not smoke before or during their visit.^20^ Another paper described the situation in Ontario, Canada where home care workers have a legal right to ask patients not to smoke in their presence and used a case study to consider in detail the ethical issues around a patient’s legal right to smoke at home and the need to protect the health of workers visiting that home.^14^

The home health and community care workforce is predominantly female. In the United States, nearly 90% of home health care workers are women.^4^ This was reflected among the studies identified: 13 reported on the gender makeup of their samples, ranging from 86% females^27^ to 95% females.^15,22,30,31^ Two studies surveyed only women health care workers’ experiences.^18,19^

Supplementary Table 2 provides detail on the fidelity assessment of each study. The table explains the methods used to assess workers’ exposure to SHS and categorizes the exposure assessment methods of each study according to the Quality Assessment Tool for Observational Cohort and Cross-Sectional Studies.^11^ All studies assessed exposure via self-report either by means of a structured questionnaire or through mention of SHS during a focus group or one-to-one interview. Reporting bias was thus likely to be high in all studies and no objective measures of exposure were identified in any study reviewed. No study assessed exposure more than once over a defined study period and there were no reports of use of validated tools or questions for the exposure assessment process. Despite the likely high variability in exposure experienced no studies reported assessment of different levels of SHS; although three studies did provide reports of different frequency of exposure.^18^,^20^,^28^

## Discussion

Despite the growing size of this occupational group internationally,^32^ there are very limited data on the number of home health or community care workers exposed to SHS during their daily work. There were no studies that reported the typical concentrations of SHS these workers encounter; and very limited and subjective data on how often subgroups of home care workers were exposed to SHS and for how long. Existing data from homes where smoking takes place shows that SHS concentrations can be considerable. Domestic rooms typically have a small volume and limited air exchange rates, SHS concentrations can be higher than the levels measured prior to smoke-free legislation in bars.^33^ Data also show that SHS remains in household air for many hours after a cigarette is smoked. Fine particulate matter measurements in over 100 homes in Scotland have shown that concentrations remain above the World Health Organization (WHO) guidance (25 µg/m^3^) for an average of 2 hours 40 minutes after a cigarette is smoked; and for over 5 hours in more than one-quarter of homes.^34^ There is a need for research to quantify the scale of this problem: how many home health or community care workers are exposed to SHS; how often and for how long; and what concentrations of SHS are experienced in individual homes and over the course of a work shift.

Our search of the literature identified three relevant guidance documents from the United Kingdom: guidance from the RCN^6^; material from the UK Health and Safety Executive^35^; and a further document from the Welsh government.^36^ The guidance provided by the Welsh government (and considered relevant for all of the United Kingdom) suggests that home health care workers should request that patients “ideally not . . . smoke for an hour or so before the visit is scheduled to take place.”^36^ The scientific basis for this 1 hour guidance figure is not given either in the Welsh government document^36^ or the RCN report.^6^

A clear pathway to protect home health and community care workers from SHS is currently lacking and consideration should be given to a global program of action that seeks to combine: (1) educating smokers about the potential harms of SHS to the visiting workers and everyone else who enters the home; (2) negotiation about what can be achieved in terms of changes to smoking behavior prior to any visit; (3) consideration of the use of tobacco substitutes such as e-cigarettes and Nicotine Replacement Therapy for a period prior to the visit; (4) limiting who is exposed – ensuring asthmatic staff, pregnant staff, or those with existing heart/lung conditions or a history of cancer accepted as being related to tobacco smoke are not exposed to SHS in any way; (5) limiting time spent in areas where SHS is present and finding areas of the home where SHS concentrations are lowest; (6) considering staff rotation so that no one member of staff is repeatedly exposed to households where SHS concentrations are highest; (7) considering legislation for smoke-free homes to protect the patient and reduce SHS exposure to all visitors, including the home health care workforce. In terms of this last point, it is worth noting that the WHO Framework Convention on Tobacco Control Article 8 guidance indicates that governments have a duty to protect individuals from tobacco smoke and that this “obligation extends to all persons, and not merely to certain populations.”^37^

From the qualitative studies identified in this review it is apparent that many workers feel a duty to put their own health at risk to deliver care and assistance to those they look after. Often smoking takes place in homes where there are multiple concerns and needs including chronic health conditions, mental health issues, and substance misuse. In these settings, workers’ exposure to SHS may be viewed as of lower priority. Some qualitative work also considers the perceived “right” of smokers to smoke in their own home.^17,19^ Social norms around protecting workers from both the acute and longer-term harms caused by SHS exposure need to change to empower workers to raise the issue with confidence and for patients to understand the need to provide those who care for them with a smoke-free environment.

The methods of assessing exposure to SHS in this group of workers were generally based around self-report with no objective measurement of exposure. The questions used in either structured survey instruments or focus group and one-to-one interviews were not available in the majority of methods sections presented for these studies. There is a need for standardized methods to quantify exposure. These will include validated questionnaire tools and also air monitoring such as fine particulate concentrations or airborne nicotine measurement. There is considerable literature on these methods for assessing occupational exposure to SHS in hospitality workers and future work could utilize some of these approaches.^38,39^

### Limitations of the Review

This was a rapid review to provide an inclusive overview of research to date and not a systematic review of studies’ findings. The searches were systematic and unrestricted by date and language; however, most of the results were single-screened for inclusion and the final set of included studies were not critically appraised although consideration was given to the fidelity of the exposure assessment methods used in each study.

The review was limited to exposure to SHS but there is increasing evidence that thirdhand smoke exposure may be harmful. Thirdhand smoke refers to tobacco-related chemicals and particulate contamination that are embedded in furnishings and carpets after smoking, and can easily be resuspended to the air or absorbed via dermal contact.^40^ Exposure to thirdhand smoke is not well understood but is likely to take place even when SHS has dissipated from the home. Similarly, we did not review exposure to e-cigarette emissions. Although these are likely to be considerably less harmful than exposure to SHS, the long-term effects of inhalation of e-cigarette emissions are unclear.^41^ Research is needed to consider the relative importance of both thirdhand smoke and e-cigarette exposure for this workforce.

## Conclusions

There is a need for further international attention on SHS exposure for workers not currently protected by existing smoke-free laws. Future policy and practice attention should be paid to taking account of the rights and responsibilities of those requiring care in their home and balancing these with the need to prevent the harmful effects of SHS to those whose jobs involve providing care and assistance in domestic settings. Research to help inform how this balance can be achieved and appropriate policies developed is required, and studies should include discussions with all relevant stakeholders, quantification of the scale of the problem, and investigations on how exposures can be best controlled in real-world settings.

## Funding

This work received no direct funding but was carried out by both authors as part of their core-funded employment at the University of Stirling. The Institute for Social Marketing is a member of the UK Centre for Tobacco and Alcohol Studies (http://ukctas.net/). Funding for UKCTAS from the British Heart Foundation, Cancer Research UK, the Economic and Social Research Council, the Medical Research Council (MR/K023195/1) and the National Institute of Health Research, under the auspices of the UK Clinical Research Collaboration, is gratefully acknowledged. The funders had no role in study design, data collection and analysis, decision to publish or preparation of the manuscript.

## Declaration of Interests

*None declared.*

## Supplementary Material

nty226_suppl_Supplementary_Table
