# Supplementary material for: Home Health and Community Care Workers’ Occupational Exposure to Secondhand Smoke: A Rapid Literature Review
Source: Nicotine Tob Res. 2018 Oct 26;21(12):1673–9. doi: 10.1093/ntr/nty226 (PMC6861833; doi:10.1093/ntr/nty226)
Supplement: nty226_suppl_Supplementary_Table [file nty226_suppl_supplementary_table.doc]

**Online supplement to: Home health and community care workers’ occupational exposure to second-hand smoke: A rapid literature review**

Angus K, Semple S

**Supplementary Table 1: Syntax for literature search strategy**

Ovid MEDLINE(R) Epub Ahead of Print, In-Process & Other Non-Indexed Citations, Ovid MEDLINE(R) Daily, Ovid MEDLINE and Versions(R) 1946 to February 07, 2018.

Run on: 12^th^ February 2018

**# Searches**

**Second-hand smoke exposure terms**

1 Occupational Exposure/

2 Inhalation Exposure/

3 Tobacco Smoke Pollution/

4 Air pollution, indoor/

5 ((occupational or environmental) adj (exposure$1 or hazard$1)).mp.

6 ((secondhand or second-hand or "second hand" or passive or involuntary or environmental or exposure or pollution) adj (smok* or tobacco or cigarette)).mp.

7 (smoke-free or smoke free or smokefree).mp.

8 ((patient$1 or client$1) adj smok*).mp.

9 1 or 2 or 3 or 4 or 5 or 6 or 7 or 8

**Home health and care workers, patients and settings terms**

10 Home Care Agencies/

11 Home Care Services/

12 Home Health Nursing/

13 Home Health Aides/

14 exp Home Nursing/

15 Homebound Persons/

16 House Calls/

17 Nurses, Community Health/

18 ((domiciliary or home) adj (care* or health* or worker* or visit)).mp.

19 house calls.mp.

20 (homebound or housebound or ((home or house) adj bound)).mp.

21 ((home or residential) adj setting).mp.

22 (private adj (home$1 or house$1 or dwelling$1)).mp.

23 (patient$1 adj (home$1 or house$1 or dwelling$1)).mp.

24 residential setting.mp.

25 ((communit* or visit* or home or "home health") adj nurs*).mp.

26 (health adj visitor*).mp.

27 10 or 11 or 12 or 13 or 14 or 15 or 16 or 17 or 18 or 19 or 20 or 21 or 22 or 23 or 24 or 25 or 26

**Final search**

28 9 and 27

Supplementary table 2: Research methods fidelity review

| **Study** | **Method of assessment of workers’ exposure to SHS** | **Different levels [concentrations or frequency] of exposure assessed** | **Exposure methods valid and reliable** | **Exposure assessed >once over time** |
| --- | --- | --- | --- | --- |
| Stephany, 1993 [9] | Simple questionnaire asking respondents to list health hazards that were part of their job | No | Question not provided; self report | No |
| Markkanen et al., 2007 [10] | Mention of SHS as a work hazard in qualitative interviews/ focus groups | No | No direct methods used; self report | No |
| Gehrs et al., 2008 [11] | Case study report describing workers entering a home where smoking takes place | No | No direct methods used; self-report | No |
| Gershon et al., 2008 [12] | Environmental contaminants experienced by workers assessed via a checklist, including exposure to ‘cigarette smoke’ | No | Question not provided. Pilot tested but not validated; self report | No |
| Sherman et al., 2008 [13] | Environmental contaminants experienced by workers assessed via a checklist, including ‘At the clients’ homes, are you or have you been exposed to cigarette smoke?’ | No | Pilot tested but not validated; self report | No |
| L'Heureux, 2009 [14] | Mention of SHS as a work hazard in qualitative interviews/ focus groups | No | No direct methods used; self report | No |
| Nabe-Nielsen et al., 2009 [15] | Questionnaire asking about ‘exposure to passive smoking’ | Yes but only in terms of frequency: almost all of the time, three-quarters of the time, one half of the time, one quarter of the time, seldom/never. | Not validated; self report | No |
| Berg et al., 2012 [16] | The focus group interview topic guide covered ‘the occurrence of SHS in the nurses’ daily work’. | No | No direct methods used; self report | No |
| Keske et al., 2013 [17] | Questionnaire asking about the number of home visits per month where the workers ‘smelled smoke’; for each in-home visit in which the worker smelled smoke, one hour of SHS exposure was assumed for calculation of monthly SHS exposure. | Yes but only in terms of an estimate of duration of exposure per month | Not validated; self report | No |
| Markkanen et al., 2014 [18] | Mention of SHS in qualitative interviews/ focus groups | No | No direct methods used; self report | No |
| Polivka et al., 2015 [19] | Mention of poor air quality in a questionnaire checklist of household hazards; mention of SHS exposure in a qualitative interview. | No | Questionnaire not validated; no direct methods used in interviews; self report | No |
| Terry et al., 2015 [20] | Semi structured one-to-one interviews on workplace health and safety: mention of SHS exposure during interview | No | No direct methods used; self report | No |
| Darragh et al., 2016 [21] | No assessment of exposure to SHS; simply considered in health and safety training | N/A | N/A | N/A |
| Hittle et al., 2016 [22, 23, 24] | Asked to recall the frequency of on-the-job environmental exposures assessed though structured interview; included hours of exposure to SHS | No | Not validated; self-report | No |
| Quinn et al., 2016 [25],[26] | Assessed indirectly using questionnaire checklist of hazards during the most recent visit, including if client smokes indoors. | Yes - the questions were repeated up to the five most recent visits with distinct clients to calculate rates of occurrence as a proportion of client visits | Piloted but not validated; self report | No |
| Wills et al., 2016 [27] | Mention of SHS in a questionnaire listing work hazards in the home setting; mention of SHS exposure in a qualitative interview prompted by ‘“Describe your experiences with hazards in the home healthcare setting”. | No | Questionnaire not validated; no direct methods used in interviews; self report | No |
| Wong et al., 2017 [28] | Questionnaire asking how often is SHS encountered in the job. | Yes but only in terms of frequency: never, rarely, sometimes, regularly, always. | Not validated; self report | No |
